# Evaluation of serum level of seven Sirtuins in Alzheimer’s Disease patients: Biomarker based on the Translational Research

**DOI:** 10.1002/alz.094968

**Published:** 2025-01-09

**Authors:** Abhinay Kumar Singh, Rashmita Pradhan, Prasun Chatterjee, Sharmistha Dey

**Affiliations:** ^1^ All India Institute of Medical Sciences, New Delhi India; ^2^ All India Institute of Medical Science, New Delhi India; ^3^ ALL INDIA INSTITUTE OF MEDICAL SCIENCES, NEW DELHI, NEW DELHI India; ^4^ All India Institute of Medical Sciences, AIIMS, New Delhi, Delhi India

## Abstract

**Background:**

Alzheimer’s disease (AD) is the most prevalent neurodegenerative disorder. Dysfunction of mitochondria and oxidative stress are known to aggravate the disease pathology. Sirtuins, NAD‐dependent deacetylases, have a well‐defined role in this pathway and thus can serve as a potential biomarker for the early detection of the disease.

**Method:**

This study evaluated the level of serum Sirtuins (SIRT1–SIRT7) in three study groups: AD, mild cognitive impairment (MCI) and geriatric control (GC) by the label free surface plasmon resonance (SPR) technology and was further validated by the Western blot experiment. ROC analysis was performed to differentiate the study group based on the concentration of serum SIRT proteins.

**Result:**

Out of seven Sirtuins, serum level of SIRT1, SIRT3 and SIRT6 (mean ± SD) were significantly decreased in AD (1.65 ± 0.56, 3.15 ± 0.28, 3.36 ± 0.32 ng/μl), compared to MCI (2.17 ± 0.39, 3.60 ± 0.51, 3.73 ± 0.48 ng/μl) and GC (2.84 ± 0.47, 4.55 ± 0.48, 4.65 ± 0.55 ng/μl). ROC analysis showed the cut‐off value with high sensitivity and specificity for cognitive impairment (AD and MCI). The concentration declined significantly with the disease progression. No specific difference was observed in the case of other SIRTs between the study groups.

**Conclusion:**

This study for the first time reports the concentration of all seven Sirtuins in serum of AD and MCI patients and reveals an inverse relationship between serum level of SIRT1, SIRT3 and SIRT6 and AD. The cut‐off values with sensitivity and specificity shows the clinical relevance of SIRT3 and SIRT6 as serum protein markers for AD.

